# Immunomodulatory Effects of MSCs in Bone Healing

**DOI:** 10.3390/ijms20215467

**Published:** 2019-11-02

**Authors:** Dalia Medhat, Clara I. Rodríguez, Arantza Infante

**Affiliations:** 1 Medical Biochemistry Department, National Research Centre, Dokki, Giza 12622, Egypt; dm.zend@nrc.sci.eg; 2 Stem Cells and Cell Therapy Laboratory, Biocruces Bizkaia Health Research Institute, Cruces University Hospital, Plaza de Cruces S/N, 48903 Barakaldo, Bizkaia, Spain

**Keywords:** MSCs, immunomodulation, inflammation, bone fractures, bone disease, cell therapy

## Abstract

Mesenchymal stem cells (MSCs) are capable of differentiating into multilineage cells, thus making them a significant prospect as a cell source for regenerative therapy; however, the differentiation capacity of MSCs into osteoblasts seems to not be the main mechanism responsible for the benefits associated with human mesenchymal stem cells hMSCs when used in cell therapy approaches. The process of bone fracture restoration starts with an instant inflammatory reaction, as the innate immune system responds with cytokines that enhance and activate many cell types, including MSCs, at the site of the injury. In this review, we address the influence of MSCs on the immune system in fracture repair and osteogenesis. This paradigm offers a means of distinguishing target bone diseases to be treated with MSC therapy to enhance bone repair by targeting the crosstalk between MSCs and the immune system.

## 1. Introduction

Bone is one of the few tissues in the human body with the ability to regenerate with a scar-free healing. Following bone injury, a complex bone healing process aimed to restore bone shape and function takes place. Defects in one or more stages of bone healing results in impaired bone repair following injury [1]. The process of bone healing involves three highly integrated and overlapping stages: inflammation, proliferation, and bone remodeling. Upon injury, the levels of inflammatory cytokines and the rate of neutrophil, monocyte, lymphocyte infiltration and macrophage polarization increase. Activated macrophages release inflammatory and chemotactic mediators, thus launching the induction of mesenchymal stem cells (MSCs) from local niches into the site of injury [2]. However, the continuous or abnormal boosting of immune cells or releasing of pro-inflammatory molecules is detrimental to the process of bone regeneration [3]. Through the proliferation phase, re-epithelialization, angiogenesis, collagen synthesis and extracellular matrix (ECM) formation take place, and, finally in the remodeling phase, collagen deposition as well as vascular maturation and regression occur [4]. In bone healing under unimpaired conditions, the immunomodulatory effect of MSCs could be crucial for establishing novel therapeutic strategies to ameliorate inefficient bone remodeling. Thus, the crosstalk between MSCs and infiltrated immune cells must be coherently modulated. From this point of view, it is necessary to understand the mechanisms that govern the relationship between immune cells and MSCs during bone repair cascades [3]. 

The strong relationship between the different stages of bone healing and immunity is well documented within the scope of osteoimmunology. In particular, the early stage of the healing process, the inflammatory stage, appears to be a vital target for immunomodulatory strategies to induce bone remodeling [3]. After injury, the pro-inflammatory response initiates the healing process, but the continuous expression of pro-inflammatory molecules has an overall negative influence on it [5]. Indeed, patients affected by chronic inflammatory conditions display an overexpression of pro-inflammatory cytokines, including tumor necrosis factor alpha (TNF-α) and interleukin-1 (IL-1), which negatively impact osteogenesis differentiation and bone formation, thus leading to bone loss [6]. Thus, a controlled inflammatory response is essential during the healing process [7], and in fact, diminishing an excessive, undamped immune response favors bone healing. 

MSCs can exert their immunomodulatory properties through two different mechanisms: direct cell contact with immune cells and by the secretion of regulatory molecules depending on the microenvironment that they face [8,9]. In the case of bone healing, this secretome can fluctuate in response to different stages of the process, targeting diverse immune cells and thus coordinating their migration, proliferation and activation [10]. In vitro experiments have shown that T cell proliferation can be suppressed by direct contact with MSCs [11]. This inhibition is driven by the interaction between the inhibitory molecule programmed death 1 (PD-1) to its ligands programmed cell death 1 ligand 1 (PD-L1) and programmed cell death 1 ligand 2 (PD-L2), both of them expressed in MSCs and in target immune cells. This engagement leads target cells to modify the expression of different cytokine receptors and signaling molecules [11]. Depending on the ratio between pro-inflammatory and anti-inflammatory cytokines present in the microenvironment they face, MSCs can secret specific cytokines such as transforming growth factor beta (TGF-β) which induces the formation of regulatory T cells (Tregs) [12]. MSCs additionally express TNF-*α*-stimulated gene/protein 6 (TSG-6), with essential anti-inflammatory functions through neutrophil migration inhibition by hampering the binding of C–X–C motif chemokine ligand 8 (CXCL8) to heparin [13]. 

## 2. Inflammation and Impact of Mesenchymal Stem Cells in Bone Healing

Fracture repair is an intricate regenerative process which can be split into direct and secondary fracture repair. Direct, primary fracture repair involves MSC migration to the site of injury, followed by their differentiation into osteoblasts and consequent secretion of ECM proteins including type I collagen, proteoglycans, and γ-carboxylated proteins that boost mineralization [14]. This takes place in types of fracture healing that occur when the fractured bone-ends are rigidly fixed and lack relative displacement, leading to little or no inflammatory response. Indirect, secondary bone fracture repair (the most common) occurs when there is no stabilization and the gap size is moderate. Thus, the repairing strategy comprises stimulatory responses within the periosteum and surrounding soft tissues, thus resulting in the formation of an external callus and subsequently stiffness improvement [15]. In this case, a complete replacing of old and damaged bone via endochondral, intramembranous ossifications, cartilaginous intermediate inflammation, cartilaginous callus formation, bony callus formation, and remodeling phase occurs [16]. 

Modulation in the inflammatory phase is fundamental for ordinary bone repair; conversely, systemic and maintained inflammation is known to be deleterious for fracture healing outcome [17]. Throughout the inflammation and in coordination with various immune responses, clot formation, tissue granulation, and cell recruitment take place. Moreover, acute bone injury is accompanied by vascular and local soft tissue damage. Meanwhile, a hematoma is formed as scaffolding for the activation of macrophages and polymorphonuclear neutrophils (PMNs) to eliminate dead cells and debris. PMNs in turn release chemokines such as chemokine ligand-2 ("CCLHYPERLINK "%22CCL2"2) and interleukin-6 (IL-6) to stimulate macrophages. However, the continuous stimulation of PMNs delays fracture repair [7].

During the first steps of bone fracture repair, both locally and systemically infused MSCs migrate to the site of injury attracted by potent chemokines released at the fracture site [18]. In this scenario, the chemokine C-X-C motif chemokine ligand 12 (CXCL12) has been found to be up-regulated [18,19,20], probably in order to promote the migration of MSCs which express C–X–C motif chemokine receptor 4 (CXCR4), the receptor for CXCL12 [21]. Moreover, recent studies have shown that the exogenous administration of CXCL12 in mouse models of bone fracture accelerated healing [22] and that migration of MSCs through the expression of CXCR4 towards the fracture area significantly improved bone healing [23].

The use of MSC-embedded porous scaffolds to directly provide cells in the fracture area to enhance bone repair has also been described; Marcacci and coworkers were the first to report promising results using autologous in vitro expanded MSCs seeded onto a porous ceramic scaffold of hydroxyapatite (HA), which perfectly fitted the bone injured areas of four patients suffering from large bone diaphysis defects [24]. After surgery, the follow up of the patients showed no adverse effects, and a perfect coupling between the implanted scaffold and the recipient bone occurred approximately half a year following surgery. 

In a model of experimental critical size femoral bone defect, Zwingenberger and colleagues implanted adenoviral induced fat tissue grafts expressing CXCL12 and/or bone morphogenetic protein 2 (BMP-2) at the site of injury. After 24 hours, MSCs were systemically injected. The migration of the injected MSCs was detected over 42 days at different times, and a marked enhancement in bone volume fraction and bone healing were observed relative to the negative control [25]. CXCL12 and BMP2 enhanced MSCs homing to the site of injury, and, moreover, osteoblasts were shown to prevail over osteoclasts, reflecting a tendency to improve bone remodeling. 

Adipose tissue is an alternate source for the isolation of MSCs. Adipose-derived stem cells (ASCs) readily provide an abundant supply, and they are safely accessible and widely suggested for tissue engineering and bone formation [26]. Both allogeneic and autologous ASCs can be successfully used in tissue remodeling without initiating a lymphocyte reaction, and, upon an appropriate stimulus, ASCs can differentiate into the osteogenic lineage [26]. ASCs have been shown to hold high osteogenic potential in experimental animal models. ASCs have shown significant ability in the repair of critical-sized calvarial defects to enhance bone remodeling in appendicular (bone that support the appendages) defects [27] and to induce spinal fusion to correct defects in small bones in murine models [28]. 

Pericytes (PSCs) are embedded in capillaries and microvasculature, presenting multipotent differentiation capacity [29,30]. In fact, it has been suggested that all MSCs are derived from PSCs due to the fact that they share identical cell surface markers [31]. Regarding their osteogenic potential, several animal models have demonstrated that PSCs can prompt powerful bone formation. Thus, during mouse incisor trauma, PSCs have been shown to be recruited to the injury site and contribute to bone repair [32]. Preclinical studies in rat spinal fusion models have shown the successful influence of delivered PSCs to induce bone regeneration, especially when performed by osteogenesis of host cells [33]. In addition, a mouse femur fracture model revealed that injected PSCs were directly involved in callus formation, suggesting that PSCs can differentiate into osteoblasts [34], and human PSCs were shown to aid bone recovery in a mouse critical-size calvarial defect model [35]. 

## 3. Mesenchymal Stem Cells Licensing as an Anti-Inflammatory Tool

The inflammatory environment to which MSCs are exposed is essential for the activation of MSCs functions. Thus, a crosstalk between MSCs and the immune system, which can be stimulated via cell–cell contact and/or by increasing the production of soluble immunomodulatory factors, is necessary to mitigate inflammation [36]. Indeed, MSCs assemble an anti-inflammatory response as result of the inflammatory cytokines secreted by immune cells [37]. This results in the production of several chemokines and growth factors by MSCs, modifying immune reactions and co-operating in tissue repair [38]. 

Ren and coworkers found that the immunosuppressive properties of MSCs are elicited by interferon gamma (IFNγ) and other pro-inflammatory cytokines such as TNFα, interleukin-1 alpha (IL-1α), or interleukin-1 beta (IL-1β) [39]. This cytokine integration induces the expression of inducible nitric oxide synthase (iNOS), a key immune suppressive molecule, and several chemokines by MSCs. The absence of the immunosuppression observed in iNOS*^−/−^* or in knockout mice for chemokine receptors such as interferon gamma receptor 1 (IFNγR1*^−/−^*) mice [39] has suggested that these chemokines drive T cell migration to MSCs, where the immune response can be inhibited by nitric oxide (NO) produced in their proximities [39]. Interestingly, wild-type MSCs have been shown to block graft-versus-host disease (GVHD) and delayed-type hypersensitivity in lethally-irradiated recipient mice but not IFNγR1^−/−^ or iNOS^−/−^ MSCs. In fact, in the case of iNOS^−/−^ MSCs, they have been shown to aggravate delayed-type hypersensitivity in mice [39]. Therefore, the cytokines involved in pro-inflammatory processes are essential to motivate MSCs’ immunosuppression effects via the collective impact of chemokines and NO.

The inflammatory cytokine interleukin 17A (IL-17A) has been shown to promote the immunosuppressive role of MSCs enhanced by IFNγ and TNFα released by activated T cells. This effect of IL-17A has been shown to be conditional on the enhanced expression of iNOS, in MSCs [40]. The AU-rich element ARE/poly(U)-binding/degradation factor 1 (AUF1), abundant in lymphoid organs, takes part in the post-transcriptional damping of inflammation-related mRNAs, a key step to diminish the immune response [41]. Interestingly, IL-17A can enhance iNOS mRNA stability through minimizing the levels of the AUF1 protein in MSCs treated with IFNγ and TNFα. Thus, AUF1 functions as a regulator through which IL-17A boosts its immunosuppressive activity on MSCs in an inflammatory environment [40]. 

MSC-induced immunosuppression seems to act in a different way according to the influence of T-cell-derived cytokines [42]. TNF-α is one such critical cytokine that is known to activate the nuclear factor kappa B (NF-κB) cascade. TNF-α-mediated NF-κB activation triggers the immune regulatory aspects of MSCs. As a matter of fact, the inhibition of either NF-κB activation or the expression of the tumor necrosis factor-alpha receptor (TNFR1) significantly abolishes the MSCs’ regulatory effect [43]. A study by Dorronsoro and coworkers demonstrated that TNF-α is effective in triggering this immune modulatory potential in human MSCs, which is in accordance with the results reported in mice, where IFN-γ seems to be a major factor [36]. Thus, the secretion of TNF-α by immune cells has been shown to induce the anti-inflammatory activity of MSC populations via the activation of NF-κB. Using models of GVDH, IFN-γ was shown to be effective in enabling MSCs to quell the immune response [44]. Interestingly, IL-1α or IL-1β alone are not able to induce a response from MSCs, but they do so in the presence of IFN-γ [39]. 

## 4. Modulation of Macrophage Polarization and MSCs-Macrophage Crosstalk

Endochondral ossification initiated after injury enhances the expansion of hematopoietic lineage cells, including macrophages. Hematopoietic cells involved in the differentiation of specific bone cell types drive MSCs to differentiate into chondrocytes or osteoblasts. Vi and coworkers reported that a lack of macrophages causes an intense decrease in ossification, suggesting that macrophages are essential not only for repair but also for normal development as well [45]. Thus, during the first stages of bone repair, under an inflammatory environment, macrophages are polarized to a pro-inflammatory M1 phenotype. M1 macrophages enhance the secretion of IL-6, TNF-α, and IFN-γ, which subsequently activate CD8+ (cytotoxic) T cells obstructing the osteogenic differentiation of MSCs. 

**Figure 1:**
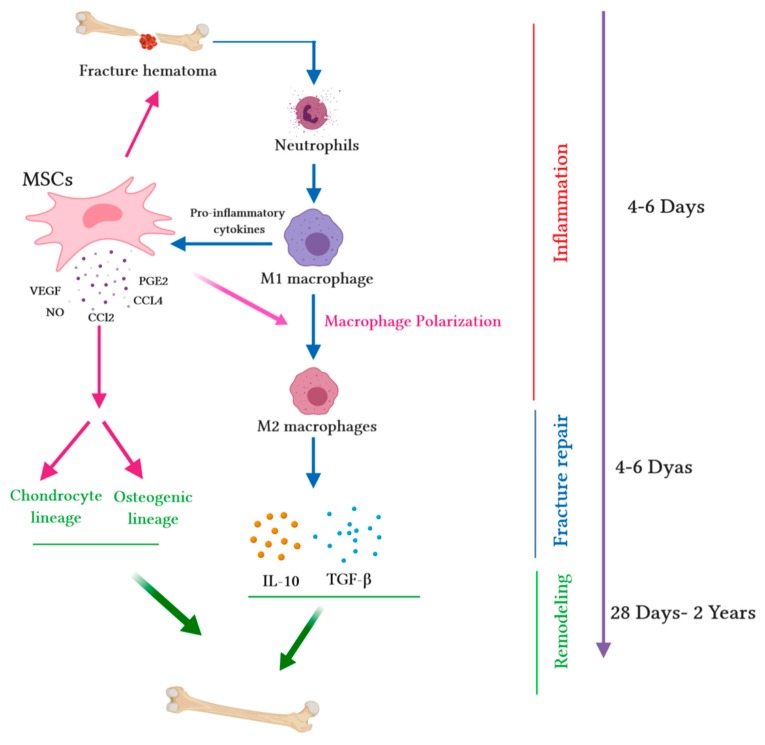
Immunomodulatory capacity of mesenchymal stem cells (MSCs) during the bone healing process. Upon fracture, a hematoma is created, and damaged blood vessels form a clot. Fracture hematoma boosts innate and adaptive immunity, especially neutrophils, resulting in the activation of macrophages. Activated M1 macrophages release pro-inflammatory cytokines and promote the migration of MSCs into hematoma. MSCs release several chemokines (chemokine ligand-2 (CCL2) and chemokine ligand-4 (CCL4)) to recruit monocytes and macrophages. Pro-inflammatory cytokines (tumor necrosis factor alpha (TNF-α), interleukin-1 alpha (IL-1α), or interleukin-1 beta (IL-1β)) stimulate MSC migration into the site of injury. During the fracture repair and remodeling stages, MSCs extremely increase the secretion of chemokines such as CCL2; mediate macrophage recruitment and polarization into the M2 phenotype; augment the secretion of vascular endothelial growth factor (VEGF), which stimulates vascularization; and secrete cytokines (such as IL-10 and transforming growth factor beta (TGF-β)), which trigger the chondrogenic differentiation of MSCs. The wide range of secretome accelerates the synthesis of the cartilaginous matrix and induces MSCs into osteogenic lineage, thus promoting intramembranous ossification at the fracture edges.

MSCs are known to deploy an anti-inflammatory effect and polarize M1 macrophages into M2 macrophages, thus modulating inflammation and launching bone repair. Maggini and collaborators indicated that MSCs turn macrophages into a regulatory profile described by a reduced capacity of inflammatory cytokines secretion and an increased capability to phagocyte apoptotic cells [46]. Anti-inflammatory factors induce bone healing processes; nevertheless, acute inflammation is remarkable for fracture repair, as it activates angiogenesis and boosts MSC migration to the site of damage [47]. M1 macrophages are known to enhance the pro-osteogenic effects of MSCs; this effect is enhanced by the transition of M1 into M2 [48] (Figure 1). Moreover, M2 macrophages are more stable than M1 macrophages, with a strong ability to modify and curtail the inflammatory response, and they are essential for tissue regeneration [49]. The activation of macrophages results in lymphocyte migration to the fracture site, launching the adaptive immune response. The consequence is the secretion of pro-inflammatory molecules such asIL-1, IL-6 and the receptor activator of nuclear factor kappa-B ligand (RANKL) among others [17]. The suppression of this inflammatory stage hampers bone repair and increases the hazard of nonunion. For example, following bone fracture, the high expression of cyclooxygenase-2 (COX-2) and prostaglandin E2 (PGE2) participate in the inflammatory phase of healing, which stimulates the differentiation of MSCs into osteoblasts [50,51]. Therefore, the inhibition of COX-2 and PGE2 by nonsteroidal anti-inflammatory drugs (NSAIDs) and selective inhibitors, both in vitro and in vivo, delays bone healing [51]. 

## 5. Paracrine Signaling Molecules of MSCs and Macrophages in Bone Fracture Healing

The interaction between MSCs and immune cells regulates both adaptive and innate immune reactions via juxtacrine and paracrine signaling [52,53]. When co-cultured with macrophages, MSCs repress the secretion of pro-inflammatory cytokines by them (TNF-α, IL-1β, and IL-6), favoring the production of anti-inflammatory cytokines (IL-10). This observation suggests that MSCs mediate this immunomodulation via iNOS- and COX-2-dependent pathways to augment PGE2 expression, which successively raises the levels of IL-10 in macrophages via the binding to prostaglandin E2 receptor 2 (EP2) and prostaglandin E2 receptor 4 (EP4) [51]. The capacity of MSCs to repress inflammatory macrophage stimulation has also been reported in a murine model, in which MSC treatment had a prophylactic effect versus lipopolysaccharide (LPS)-induced septic shock through the modulation of macrophages and neutrophils into a more anti-inflammatory phenotype [54]. MSCs in a transwell co-culture with macrophages markedly prohibited the polarization of M1 macrophages and motivated the polarization of M2 macrophages [55]. Comparable findings have been reported with human peripheral blood monocytes, which have been shown to stimulate the development of M2-like phenotype macrophages when co-cultured with MSCs [56]. Moreover, the exposure of human MSCs to IFN-γ and TNF-α has been shown to increased levels of indoleamine 2, 3-dioxygenase (IDO), which participates in the polarization of monocytes into IL-10-secreting M2 macrophages and indirectly represses the proliferation of T cell [57].

MSCs also regulate macrophage chemotaxis. Thus, compared to human fibroblasts and mouse bone marrow MSCs (BM-MSCs), hMSCs secrete a wider range of chemokines, mainly chemo attractants for monocytes and macrophages such as chemokine C–C ligand 2 (CCL2) and chemokine C–C ligand 4 (CCL4) [58]. The interaction between IFN-γ with other pro-inflammatory cytokines including TNF-α, IL-1α, and IL-1β stimulates MSCs in injured tissues; activated MSCs enhance the secretion of several chemokines and mediate the immunomodulation of infiltrated macrophages, potentially reinforcing tissue regeneration [59].

Vi and coworkers used transgenic mice to deplete macrophages to investigate macrophage adequacy in bone development, growth, and repair [45]. They showed that bone union was ineffective when macrophages were depleted—calluses were fibrotic, smaller and contained less bone. This result demonstrated that macrophages are critical for fracture repair by inducing the osteogenesis of MSCs. In addition, a previous study demonstrated that osteal macrophages (osteomacs) not only participate in intra-membranous bone healing but are also targets for primary anabolic bone therapies [60].

Studies in vitro and in vivo have investigated the functions of these bone macrophages in osteoblast differentiation by producing bone morphogenetic proteins (BMPs) [61] and oncostatin M [62]. In addition, it has been reported that a decrease of bone macrophages prohibits the differentiation of MSCs into primary osteoblasts [63]. In vivo, the selective removal of osteal macrophages, but not osteoclasts, has confirmed that the absence of osteal macrophages could be a main cause in the reduction of bone formation, as well as a cause of defects in bone growth in young mice and osteoporosis [45]. In vivo experimental models of femoral fractures have shown the impact of depleting macrophages by clodronate liposome treatment during the different bone healing stages [64]. No serious effects derived from macrophages mitigation were observed on the early fracture healing stage. However, a lack of macrophages caused delays in the genesis of hard calluses, thus severely altering endochondral ossification. Treatment with clodronate liposomes caused the late bone unification of cartilage and promoted periosteal bone formation. During the healing process, Schlundt and collaborators evaluated M1 and M2 macrophage subsets in non-treated mice, and they noticed that M2 macrophages prevailed during the ossification stage, suggesting that the boosting of the M2 phenotype in macrophages is critical in bone healing. In addition, they reported that the induction of the M2 macrophages by IL-4 and IL-13 markedly stimulated bone formation over 21 days of investigation. Taken together, it is clear that balance in M1/M2 macrophage function seems compulsory for fracture healing and successful regeneration.

Seebach and coworkers reported that the implantation of MSCs at the site of experimental bone defects in a hydrogel induced an increase in the expression of vascular endothelial growth factor (VEGF). They noticed that the prompt infiltration of M1 macrophages and endothelial cells ameliorated vascularization and bone remodeling in the area of injury [58]. In accordance with previous results, it has been reported that MSCs implanted into murine cranial defects differentiated into osteoblasts, evoked macrophage polarity, and stimulated repair [65]. These reports suggest that MSCs regulate the chemotaxis and activities of macrophages. Moreover, MSC-derived factors positively engaged in bone reconstruction through the modification of the functions of macrophages. 

## 6. Regulatory Effects of MSCs on T Lymphocytes in Bone Fracture Repair

Certain cells of the adaptive immune response, such as CD8+ T cells, can have a detrimental effect on bone healing if they prolong the secretion of pro-inflammatory factors [66]. On the contrary, Tregs are known to positively impact on fracture healing due to the fact that they favor the osteoblast differentiation from MSCs by inhibiting the secretion of pro-inflammatory cytokines via activated T cells [67]. Through direct cell–cell contact [39] or paracrine secretion [68], MSCs play a dual role in modulating these different subsets of T cells in bone healing. On one hand, MSCs can inhibit T cell (in both CD4+ and CD8+ subsets) activation and proliferation, as shown by in vitro mixed lymphocyte reaction [69,70] by inducing the G0 arrest of the cell cycle [71]. Moreover, MSCs stimulate the apoptosis of T cells by the Fas/FasL-dependent pathway [72] and by the secretion of PD-L1 [73]. On the other hand, MSCs can both induce the formation of CD4+CD25+ Foxp3+ Tregs [74,75] and trigger Tregs’ immunosuppressive abilities through the secretion of heme oxygenase-1 (HO-1) and IL-10 and the upregulation of PD-1 receptors on Tregs [76].

## 7. MSCs Derived Exosomes in Bone Fracture Repair

Exosomes are tiny (30–120 nm) extracellular vesicles (EVs) originating from the plasma membrane that are secreted from different cells into most human fluids. They contain a mixture of different molecules: nucleic acids, proteins, metabolites, and lipids, thus mediating intercellular communication in both ordinary and pathological conditions [77].

The output of exosomes from MSCs has been shown to be immense when compared to other cell types. Additionally, many regenerative characteristics of stem cells have been shown to be regulated through secreted exosomes [78]. Moreover, MSC-derived exosomes (MSC-DEs) ameliorate the repair of damaged tissues and are also involved in the modification of immune responses through differentiation, paracrine signals, and other secreted molecules such as microvesicles [79]. MSCs express trophic factors including growth factors, cytokines and chemokines that participate in different cell activities [80]. In fact, MSC paracrine signaling is achieved by cytokines and chemokines with anti-apoptotic, anti-inflammatory, anti-oxidative, and pro-angiogenic characteristics [81]. Furuta and coworkers showed that both an MSC-conditioned medium (CM) and MSC-DEs accelerated fracture healing, not only through the induction of MSCs or progenitor cells by cytokines (CCL2 and CXCL12) but also through the enhancement of osteogenesis and angiogenesis [82]. Indeed, these mechanisms are arranged by microRNAs (miRNAs) in exosomes, which control tissue development and homeostasis via fine-tuning gene expression [82]. In addition, exosomal angiogenic factors (VEGF and IL-6) enhance bone growth and fracture healing by stimulating endothelial cells [83].

miRNA in MSC-DEs may participate in fracture healing and tissue regeneration [84]; for example, exosomal miR-21 (anti-apoptotic miRNA) was found to be highly expressed in MSC-DEs [85]. MiR-21 promotes the osteogenic differentiation of MSCs [85], and the local injection of MSCs overexpressing miR-21 has been shown to improve fracture healing in an experimental rat model [86]. In addition, three other miRNAs (miR-4532, miR-125b-5p, and miR-338-3p) have been shown to be highly expressed in MSC-DEs, suggesting that these miRNAs play a critical role in fracture healing [82]. 

## 8. Immunomodulation as a Mechanism in MSC-Based Therapies for Bone Diseases

**Figure 2:**
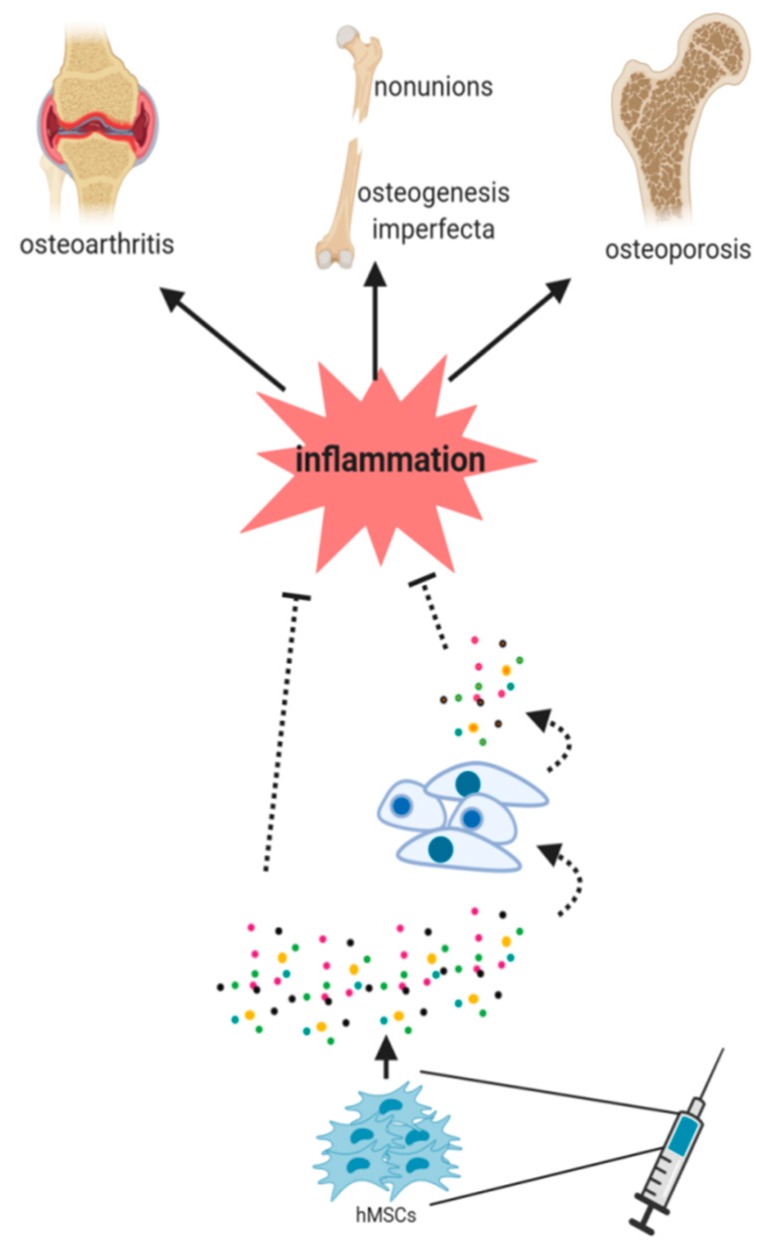
hMSCs should face an inflammatory microenvironment to exert their benefits in cell therapy purposes. The paracrine signaling of hMSCs has been postulated to be an essential mechanism in this healing process. The secretion of factors by MSCs (paracrine signaling) seems to be essential in this process. However, it is still unknown (dash lines) whether the secreted factors directly induce the healing or if they induce different signaling pathways in the host cells, thus stimulating then tissue regeneration.

Currently, patients considered for MSCs treatments are typically refractory to all conventional therapies and/or suffer a low prevalence disease. Consequently, many of these clinical trials are performed with a small number of patients, making it difficult to reach statistically significant conclusions. There has been mounting evidence pointing towards the idea that the paracrine secretion of MSCs mainly orchestrates the beneficial effects observed in cell therapy. In fact, after facing the local environment, MSCs secrete a plethora of bioactive factors that affect the biology of host cells [52]. However, the molecular mechanisms responsible for these effects remain elusive. Thus, two scenarios (not mutually exclusive) are being considered to explain this effect: (1) The improvements of MSC therapy could be directly induced by their secreted factors, and/or (2) these factors could be the responsible for the activation/repression of different signaling pathways in the resident cell population which then stimulates host tissue regeneration (Figure 2). With respect to the immunomodulatory properties of MSCs, it should be noted that these depend on two factors: the inflammatory environment to which MSCs are exposed and the tissue of origin of MSCs. These observations imply that before expecting success with MSC therapies, a suitable inflammatory disease to be treated must be selected, as should an appropriate source of MSCs [87]. Regarding bone diseases, in this review, we have focused on bone defects someway connected to an inflammatory process which are currently treated or suitable to be treated with MSC therapies.

### 8.1. Nonunion Bone Fractures

As mentioned previously, fracture healing usually occurs during the first six-to-eight weeks after an injury and encompasses three stages: inflammation, repair and remodeling [7]. Nonunion fractures are complications that imply a permanent failure of healing six months after a bone fracture occurs [88]. Though the physiopathology of nonunions remains unclear, there are risk factors associated with this condition, such as diabetes and age, both of which are considered to be chronic inflammatory stages [7]. In an effort to understand the molecular and cellular mechanisms leading to nonunions, some research has focused on the in vitro study of the MSCs isolated from affected patients. These studies have reported that the osteogenic potential of MSCs from nonunion patients is unaffected [89,90]. However, a decrease in proliferation has been observed in the case of bone BM-MSCs from these patients [89]. Remarkably, changes in the serum levels of chemokines and growth factors have been described in nonunion patients, e.g., a significant increase in the expression of IL6, a known pro-inflammatory cytokine, which stimulates the migration of MSCs to the site of bone injuries [48]. Several studies have discussed the efficiency of BM-MSCs in augmenting nonunion. Connolly and coworkers percutaneously injected BM-MSCs into the defect site in 20 patients with tibial nonunion; interestingly, 18 of them accomplished union in 6–10 months [91]. Similar studies have shown comparable results [92,93]. In addition, Ismael and collaborators compared patients infused with ex vivo expanded autologous BM-MSCs (14–18 × 10^6^) with those receiving autograft iliac crest transplantation [94]. Though all the patients showed effective unification within a year, the patients treated with BM-MSCs presented faster clinical improvements. Thus, BM-MSCs provide an efficient approach for treating nonunion. As mentioned above, the initial stages of bone healing include an acute inflammatory response in which mainly macrophages release cytokines, chemokines and growth factors to recruit additional inflammatory cells and to induce MSC migration to the site of injury. However, if the inflammatory response becomes chronic, the healing process is hampered [95]. Considering that a risk factor of suffering nonunions is diabetes or age, both chronic inflammatory conditions, and the increase levels of IL-6 detected in serum from nonunion patients, it is likely that an increased inflammatory process could be partially governing the pathophysiology of nonunions. Encouraged by the in vitro finding that osteogenic capacity of MSCs in nonunions is not affected, a recent prospective study treated nonunion patients with autologous expanded BM-MSCs [96]. Fracture union was observed in 21 patients from a total of 35 receiving cell therapy, and a faster in vitro MSC doubling time predicted the positive outcome of nonunions. Though the authors did not study the pro-inflammatory cytokine levels before and after the cell therapy, a modulation of macrophage polarization towards an anti-inflammatory phenotype could be a possible mechanism of MSC therapy in nonunions. Future studies addressing this observation should clarify the role of an increased inflammatory response in nonunion patients and the possibility of counteracting it with MSC therapy. 

### 8.2. Osteoporosis

Osteoporosis is a common age-related disorder, resulting from a switch to bone resorption at the expenses of bone formation, that affects bone remodeling. This hampered bone remodeling leads to low bone mass and the micro-architectural deterioration of bone, increasing the risk of fractures. Patients affected by chronic systemic inflammatory diseases such as rheumatoid arthritis, inflammatory bowel disease or systemic lupus erythematosus also have an increased risk of secondary osteoporosis [97]. Thus, MSC therapy approaches for these patients suffering chronic inflammatory diseases and osteoporosis could provide a potential new therapeutic strategy, although so far there have been no clinical trials of MSC transplantation in osteoporosis. Preclinical studies in animal models of osteoporosis have shown that MSC administration may improve bone mineral density (the most reliable predictor of fracture risk), thus suggesting a possible beneficial effect of cell therapy [98]. The immunomodulatory effects of MSCs could be responsible for this positive outcome, but these studies did not focus on the mechanisms governing MSC effects. While these results are encouraging, further data from basic research studies as well as from preclinical studies are needed to understand the mechanisms underlying MSC therapy for osteoporosis before proceeding with human clinical studies.

### 8.3. Osteogenesis Imperfecta

Osteogenesis imperfecta (OI) is a rare skeletal dysplasia that affects 1 in 20,000 births. Clinically and genetically highly heterogeneous, it is characterized by a decreased bone mass and altered microarchitecture that results in an increased bone fragility [99]. OI is usually caused by autosomal dominant mutations in the genes encoding procollagen alpha chains (*COL1A1*/*COL1A2*), which may cause defects in collagen quantity (a milder form of OI) or in collagen quality due to the abnormal assembly of the protein (moderate-to-severe-to-lethal forms of OI) [100]. Other severe forms of OI are due to recessive mutations in non-collagenous genes that affect collagen post-translational modifications, bone matrix mineralization, and osteoblast differentiation and function [101]. There are no curative interventions for OI patients. MSCs have been used as cell therapy for patients affected by OI, with the expectation of replacing target tissue (bone) with infused MSCs. In 2002, the first transplantation of allogenic adult MSCs into six OI pediatric patients was performed [102]. Despite the fact that MSC engraftment was quite low (< 1% in osteoblasts), a short-term improvement of the linear growth velocity of patients was appreciated. Later, this research group demonstrated chondrocyte proliferation in mice after infusing secretome from MSCs, thus suggesting that the paracrine secretion of MSCs could be responsible for the clinical improvement observed in patients [103]. The prenatal transplantation of human fetal MSCs also has been shown to be safe and effective in two fetuses affected by OI. The rationale of infusing MSCs intra-utero was that the cell administration before birth should more effectively alleviate OI symptoms [104]. However, these studies have demonstrated that a single transplant of MSCs appears to be insufficient; therefore, subsequent MSCs infusions seem to be needed to maintain the observed clinical benefits. Interestingly, recent works have shown that inflammation could be present in OI children, a fact unknown until now. Thus, a cohort of children affected by moderate–severe OI was shown to have increased platelet counts, although no other pro-inflammatory signs were detected [105]. Interestingly, the fractures were associated with a higher platelet counts rather than OI illness per se. In line with this observation, increased levels of the C-reactive protein (C-RP) and erythrocyte sedimentation rate (ESR) have been recently reported in OI type V patients suffering the typical hyperplastic callus formation, a distinctive clinical manifestation of this type of OI [106]. Moreover, a whole transcriptome analysis in mice models of OI type V has also suggested the potential activation of inflammatory pathways in these mice [107]. All in all, these findings point to an underlying role of inflammation in the pathophysiology of OI and provide an important support of the rationale of using MSC therapy for OI treatment. Thus, the observed benefits of MSC therapy in OI could be in part due to the immunomodulatory capacities of the infused MSCs once they face the inflammatory environment to which they are exposed in OI. 

### 8.4. Osteoarthritis

Though not exclusively restricted to bone tissue, osteoarthritis (OA), a complex joint disease, is characterized first by a loss of cartilage which then, due to abnormal joint loading, leads to subchondral bone abnormalities driven by excessive bone remodeling. Macrophages are elevated in OA joints, contributing to synovitis and fibrosis, both hallmarks of OA [108]. As mentioned above, the ability of MSCs to polarize macrophages into inflammation-resolving subtypes [52] makes OA a potential candidate to be treated with MSC therapy. Clinical trials using the intra-articular injection of either allogeneic or autologous bone marrow-derived MSCs in knee OA have reported statistically significant clinical improvements in terms of pain and reduced synovial inflammation [109,110]. Notably, a substantial dose-response effect has been detected in the majority of these works, and higher doses (40–50 × 10^6^ cells) have reported better outcomes. In the case of adipose-derived MSCs, only the highest doses of cells (100 × 10^6^ MSCs) has shown efficacy, reflecting MSC tissue-specific differences in dose efficacy [111]. However, in 2016, a study using pooled, allogeneic MSCs showed improved outcomes in the pain measurement scores in patients receiving a low dose of cells (25 x 10^6^ MSCs), possibly due to the fact that following cells injection, hyaluronic acid was administered as a supporting matrix [112]. Regarding the in vivo mechanism of action of MSC cell therapy in OA, a recent study showed a decrease in pro-inflammatory markers in the synovial fluid from patients (such as the levels of IL-12p40 and the CD14+CD16+ monocyte/macrophage subsets) three months after BM-MSC injection, supporting an anti-inflammatory mechanism of action of MSCs [110]. Strikingly, Chahal and coworkers performed a molecular fingerprinting of the licensed MSCs coming from the 12 patients enrolled in their study, and they showed that an increased expression of anti-inflammatory and anti-fibrotic molecules in the MSCs was predicted to produce better clinical outcomes in patients. The relevance of this study is twofold: First, an immunomodulatory mechanism of MSCs is suggested to be driving the beneficial effects of MSC-based therapy, and second, the gene expression profiling of donor MSCs opens the door to prospective MSC molecular screenings to select the cells with the best potential to improve the effects of MSC-based cell therapies. 

## 9. Conclusions

Compelling evidence supports an important role for MSCs in the bone healing process, due mainly to their immunomodulatory abilities through the secretion of paracrine factors. Clinical trials have validated their safety and effectiveness as well as their potential use with cell therapy purposes for bone diseases with an underlying inflammatory condition. However, despite the great progress made since the discovery and characterization of MSCs, it is necessary to evaluate the osteogenic potential of MSCs for bone recovery and the regulation of inflammation through the selection of those donors and/or subpopulations of high osteogenic capacity. Additional work will clarify the mechanisms triggered by the immunomodulation of MSCs to enhance bone repair. 

## Abbreviations

MSC	Mesenchymal stem cells
ECM	Extracellular matrix
TNF-α	Tumor necrosis factor alpha
IL-1	Interleukin-1
PD-1	Programmed death 1
PD-L1	Programmed cell death 1 ligand 1
PD-L2	Programmed cell death 1 ligand 2
TGF-β	Transforming growth factor-β
Tregs	Regulatory T cells
TSG-6	TNF-α-stimulated gene/protein 6
CXCL8	C-X-C motif chemokine ligand 8
PMNs	Polymorphonuclear neutrophils
CCL2	Chemokine ligand-2
IL-6	Interleukin-6
CXCL12	C-X-C motif chemokine ligand 12
CXCR4	C-X-C motif chemokine receptor 4
BMP-2	Bone morphogenetic protein 2
ASCs	Adipose-derived stem cells
PSCs	Pericytes
IFNγ	Interferon gamma
iNOS	Inducible nitric oxide synthase
GVHD	Graft-versus-host disease
NO	Nitric oxide
IL-17A	Interleukin 17A
AUF1	ARE-binding protein ARE/poly(U)-binding/degradation factor 1
NFκB	Nuclear Factor Kappa B
TNFR1	Tumor necrosis factor-alpha receptor 1
RANKL	Receptor activator of nuclear factor kappa-B ligand
COX-2	Cyclooxygenase-2
PGE2	Prostaglandin E2
IL-10	Interleukin 10
LPS	Lipopolysaccharide
IDO	Indoleamine 2,3-dioxygenase
BM-MSCs	Bone marrow-derived mesenchymal stem cells
VEGF	Vascular endothelial growth factor
HO-1	Heme oxygenase-1
EVs	Extracellular vesicles
MSC-Des	Mesenchymal stem cell-derived exosomes
CM	Conditioned Medium
LD	linear dichroism
miRNA	Micro RNA
OI	Osteogenesis imperfecta
C-RP	C-reactive protein
ESR	Erytrocyte sedimentation rate
